# Capturing Complex Vaccine-Immune-Disease Relationships for Free-Ranging Koalas: Higher Chlamydial Loads Are Associated With Less IL17 Expression and More Chlamydial Disease

**DOI:** 10.3389/fvets.2020.530686

**Published:** 2020-09-25

**Authors:** David Lizárraga, Peter Timms, Bonnie L. Quigley, Jon Hanger, Scott Carver

**Affiliations:** ^1^School of Natural Sciences, University of Tasmania, Hobart, TAS, Australia; ^2^Genecology Research Centre, School of Science and Engineering, University of Sunshine Coast, Sippy Downs, QLD, Australia; ^3^Endeavour Veterinary Ecology Pty Ltd., Toorbul, QLD, Australia

**Keywords:** structural equation model, *Chlamydia*, vaccine, koala, cytokine

## Abstract

**Background:** Chlamydial disease is a major factor negatively affecting koala populations. Vaccination is a promising management option that would result in immune-mediated protection against disease. Measuring and assessing vaccine efficacy can be challenging owing to both direct and indirect interactions caused by vaccination. In this study, we investigate vaccine-immune-chlamydial load-disease relationships from MOMP (major outer membrane protein) vaccine trials to protect healthy free-ranging koalas against *Chlamydia*-related diseases.

**Methods:** We created *a priori* hypotheses based on data sources and perceived direct and indirect interactions from koalas vaccinated 6 months prior. Each hypothesis was tested as a structural equation model separately for either the urogenital or the ocular site to evaluate possible causality among measured variables. Model averaging was used as multiple models fit the data, and the strength of relationships was examined through averaged coefficients and the raw data.

**Results:** We found more relationships in urogenital models as compared to ocular models, particularly those with interleukin 17 (IL17) mRNA expression compared to models with interferon gamma (IFNγ) expression. In the averaged model with IL17, urogenital chlamydial load was positively associated with disease and negatively associated with IL17 expression. MOMP vaccination had a trending effect for reducing urogenital chlamydial load and also had a strong effect on increasing IL17 expression. Not surprisingly, urogenital chlamydial load was a positive predictor for the development of urogenital disease at 6 months post-vaccination.

**Conclusions:** Despite multiple potential sources of variation owing to the koalas in this study being free-ranging, our analyses provide unique insights into the effects of vaccinating against *Chlamydia*. Using structural equation modeling, this study has helped illuminate that the expression of the immune cytokine IL17 is linked to MOMP vaccination, and animals with a high urogenital chlamydial load expressed less IL17 and were more likely to develop disease, enhancing previous investigations. Going beyond univariate statistics, the methods used in this study can be applied to other preclinical vaccination experiments to identify important direct and indirect factors underpinning the effects of a vaccine.

## Introduction

Koala (*Phascolarctos cinereus*) populations, particularly in the Australian Capital Territory and in the Australian states of New South Wales and Queensland, have suffered staggering losses over recent years (1–4) leading to their conservation status being listed as “vulnerable” per the IUCN (5). A number of factors negatively affect koala populations including: habitat loss (1, 6), climate change (7), bushfires (8), motor vehicle accidents (6), dog attacks (8), and disease (9). Deterministic age structured matrix models (9) of four of these factors (habitat loss, dog attacks, motor vehicle collisions, and disease) indicate that reducing the prevalence of disease may stabilize koala populations. The magnitude of disease-related mortality within a given population is potentially exacerbated by environmental stressors including climate change, habitat loss resulting from urbanization, and environmental disasters such as bushfires, though to our knowledge no study has investigated this in wild koala populations (10). Amid its variable magnitude, the reduction of disease has been a key management strategy of this species (2, 9, 11, 12).

*Chlamydia* is an obligate, intracellular bacterium that is the most prevalent disease-causing pathogen in wild koalas (12, 13). Koalas are hosts to two bacterial species of *Chlamydia* (*C. pecorum* and *C. pneumoniae*), however, modern vaccine candidates target the more prevalent species, *C. pecorum*, that typically infects epithelial cells in the ocular and urogenital mucosa (14). Sterility and disease-related mortality as a result of chlamydial infections have a direct, negative impact on koala population dynamics and are relatively common among some free-ranging koala populations (11, 15). Chlamydial infections in koalas are treatable with antibiotics, but this management strategy is potentially fatal for the specialized microflora in the koala gastrointestinal tract that is necessary for eucalyptus digestion (16). Vaccinating koalas is a promising tool for disease management that is modeled to have clear benefits, particularly at the population scale. Modeling by Craig et al. (17) suggested that chlamydial vaccination could stabilize koala populations after 5 years of using a vaccine (with protective efficacy of 75%) administered to around 10% of koalas per year. Such a vaccine does not yet exist, however, many studies within the last decade have advanced the development of a vaccine for koalas against *Chlamydia*.

Much of the foundational work for chlamydial vaccine development has used captive koalas under controlled veterinary conditions [reviewed by Phillips et al. (18)]. However, field trials that encompass a greater range of natural variables provide a more accurate picture of vaccine efficacy. To date, there are two published field trials testing a chlamydial vaccine using free-ranging koalas. The first study was by Waugh et al. (19). This vaccine consisted of three chlamydial major outer membrane proteins (MOMP) from three genotypes (A, F, and G) and an Immunostimulating Complex (ISC) adjuvant, that was delivered subcutaneously three times over 3 months (given at 1-month intervals). Chlamydial load and disease were compared 6 months post-vaccination between vaccinated and control koalas in the 60 koala trial. Vaccinated animals had increases in anti-*Chlamydia* immunoglobulin G (IgG) and lower levels of chlamydial load and prevalence of disease (at both the ocular and urogenital sites) compared to control koalas 6 months after the initial vaccination. The second field study was by Desclozeaux et al. (20). This trial tested two vaccine formulations consisting of either MOMP (three genotypes A, F, and G) or PMP (peripheral membrane protein, genotype G). Both vaccines were delivered subcutaneously alongside a tri-adjuvant (PCEP, IDR1002, and polyI:C) and vaccinated groups were compared to non-vaccinated control koalas (21 koalas in each group, 63 in total). Both MOMP and PMP vaccinated koalas had elevated interferon gamma (IFNγ) and interleukin (IL) 17 mRNA expression 6 months post-vaccination compared to pre-vaccination levels. Additionally, koalas vaccinated with MOMP had lower chlamydial loads compared to control koalas 6 months post-vaccination. Combined, these field trials have examined enough koalas that, for the first time, more detailed modeling analysis of factors involved in vaccine responses in koalas can be considered.

The koala immune response has been a focal point of research in recent years, particularly to chlamydial infection [reviewed by Madden et al. (21)]. Several important cytokines or antibodies in response to chlamydial infection in koalas have been identified: IFNγ, IL17, IL10, tumor necrosis factor alpha (TNFα), IgG, and IgA. As multiple aspects of the koala immune response are poorly understood, researchers often refer to vaccine trials against two other chlamydial species, *C. trachomatis* or *C. muridarum*, where mice are most often used as host models of infection (22, 23). Of these murine trials, the most commonly measured host cytokine in response to chlamydial infection is IFNγ (22), where the expression of IFNγ has been associated with protection against chlamydial disease (24). Increases in IFNγ concentration *in vitro* can lead to the degradation of tryptophan, leading to the starvation of *C. trachomatis* of this essential amino acid and inducing chlamydial persistence [an inactive, intracellular pathogen response to external stressors; (25)]. A recent study has shown *C. pecorum* to be resistant to increasing concentrations of IFNγ *in vitro*, suggestive of different immune-evasion mechanisms as compared to *C. trachomatis* (26). This difference from *C. trachomatis* (which is sensitive to IFNγ) could lead to a different effect of IFNγ in koalas to *C. pecorum* infection. Lastly, in murine hosts, elevated levels of both IFNγ and IL17 increased the production of inducible Nitric Oxide synthase (iNOS), promoting the production of microbicidal nitric oxide (NO) that correlated with the reduction of chlamydial load (27). Some studies, however, have suggested that an elevated iNOS response to infection (elevated by host cytokines) may be associated with scarring of the fallopian tubes and immunopathogenesis (28, 29). The relationships between chlamydial vaccination, the host immune response (particularly IFNγ and IL17), chlamydial load, and disease is still poorly understood in koalas.

Clearly, a key challenge in chlamydial vaccine research is understanding complex direct and indirect immune-mediated control of infection and disease. In this study, we aimed to model important direct and indirect factors surrounding the vaccination of free-ranging koalas: vaccination status, host immune parameter, chlamydial load, and disease. We used structural equation models to identify the directionality and magnitude of direct and indirect relationships when all four variables are modeled together. This statistical method has been used previously to identify both multiple environmental and individual factors that affect chlamydial disease pathology in koalas (30). We modeled vaccination status, the expression of an immune parameter (either IFNγ or IL17), chlamydial load, and disease status at two important mucosal sites (ocular and urogenital) using data collected by Waugh et al. (19) and Desclozeaux et al. (20). We tested six hypotheses to (1) identify the relationships between immune parameter and chlamydial load, and (2) identify the relationships between host immune parameter and disease.

## Methods

### Experimental Design

Following vaccination or non-vaccination, control and vaccinated koalas were resampled at an average of 5.95 months (± 0.33, 95% CI, referred to as 6 months post-vaccination hereafter). We adopted a cross-sectional comparison of control to vaccinated free-ranging koalas to determine if our vaccines against *Chlamydia* was effective, given uncontrolled background variation of individual and immune factors. The intent was to assess whether vaccination superseded background individual variation (i.e., immune history, genetics, and seasonality) in parameters, which we know does not protect koalas against *Chlamydia* disease. We note that koalas were sampled across seasons (Spring *n* = 17, Summer *n* = 3, Autumn *n* = 9, Winter *n* = 11), but in preliminary analyses we found no effect of season on measurements of either IL17 (Kruskal Wallis *X*^2^ = 1.060, *p* = 0.787) or IFNγ (Kruskal Wallis *X*^2^ = 2.185, *p* = 0.535).

### Pooling Data From Both Field Trials

**Figure 1:**
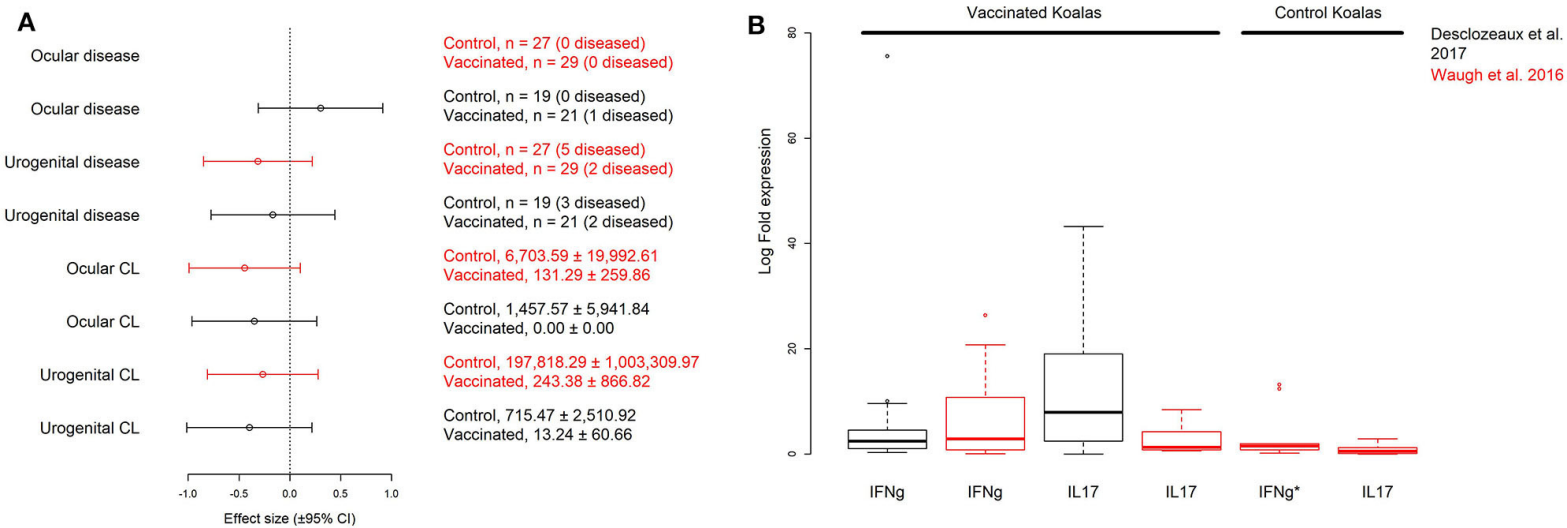
Comparison of hedge's g (± 95% CI) effect sizes for disease and chlamydial load (CL) data collected at the urogenital and ocular sites for vaccinated and control groups **(A)**, and log_10_ transformed interferon γ (IFNg) and interleukin 17 (IL17) cytokine expression for vaccinated and control groups **(B)** of wild koalas in two published MOMP-vaccination trials (see Appendices 1, 2): Desclozeaux et al. (20) (black effect sizes and boxplots) and Waugh et al. (19) (red effect sizes and boxplots). All measurements were taken 6 months post-vaccination. Sample size (with n diseased) or mean (± S.D.) copies/μL are shown for each factor in this (A). Despite differences between the two trials, we found no evidence against pooling data between the two trials after performing a meta-regression comparing the effect of MOMP vaccination on disease and chlamydial load comparing treated and control koalas (meta-regression of effect size, g by field trial, *p* = 0.892). Immune cytokine measurements (fold gene expression relative to the housekeeping gene GAPDH) from vaccinated koalas 6 months post-vaccination were comparable between the two studies after performing a non-parametric Wilcoxon rank sum test for IFNg or IL17 (*p* = 0.936, and *p* = 0.075, respectively). To better visualize the data, the IFNg expression from one control koala (IFNg*) is excluded from the figure (2592.26 log fold expression), but included in all analyses. Only Waugh et al. (19) measured the expression of both IFNg and IL17 from non-vaccinated control koalas. Additionally, no effect size could be estimated for ocular disease in the trial by Waugh et al. (19) as all animals were clinically healthy at the ocular site.

Previously collected data from two field trials of free-ranging koalas vaccinated against *Chlamydia* described by Waugh et al. (19) and Desclozeaux et al. (20) were used in our analysis (see Appendices 1, 2 for a summary of the methods for both studies). The two field trials (different vaccination schedules and different adjuvants) were investigated to determine if differences exist in the data for MOMP vaccinated individuals (PMP vaccinated individuals were excluded). We first used a meta-analysis to compare the effects of vaccination (control vs. vaccinated animals) on chlamydial load and disease prevalence at either the ocular or urogenital site for 6 month post-vaccination data between studies (Figure 1A). In one trial (19), all koalas (vaccinated and control; see Figure 1A) remained healthy at the ocular site (no effect size could be calculated). An effect size (or a Hedge's *g*) and variance were calculated to estimate the effect of MOMP vaccination on chlamydial load (at the ocular and urogenital sites) and disease (at the urogenital site) for both field trials [a total of 6 effect sizes; see Borenstein et al. (31)] (19, 20). A meta-regression was performed on the effect sizes using the MAd package (32) in the statistical program R [v3.5.3; (33)] and an *I*^2^ statistic was calculated to determine heterogeneity between studies for each measurement (as omnibus models) (31). As control koalas in the field trial by Desclozeaux et al. (20) did not have measurements of IFNγ or IL17 expression, a comparison was made for these immune measurements using univariate methods (Figure 1B). As no evidence indicated differences between the two trials, the data collected from Waugh et al. (19) and Desclozeaux et al. (20) were pooled to test hypothesized models with chlamydial load, disease, and immune response measurements taken at 6 months after the first chlamydial vaccination.

### Creation and Testing of Hypothesized Models

Six *a priori* structural equation models were created based on biological hypotheses proposed in the literature (Table 1; see Appendix 1 for additional background information and Appendix 2 for measurement sample sizes). We created our hypothesized models with the assumptions that vaccination affected chlamydial load and host immune parameters (black arrows between V. Status and either C.L. or I.P., see Table 1) established in the original publications by Waugh et al. (19) and Desclozeaux et al. (20). We also made our hypothesized models with the assumption that MOMP vaccination was not directly associated with disease based on previous studies that evaluated the safety of vaccinating healthy koalas against *Chlamydia* (34, 35). Lastly, we assumed that there was a link between chlamydial load and chlamydial disease (black arrow between C.L. and Disease, see Table 1) based on structural equation models of non-vaccinated free-ranging koalas (30).

**Table 1:** Six hypotheses of MOMP (major outer membrane protein) vaccination status (V. status), immune parameter (I.P.) mRNA expression, chlamydial load (C.L.), and disease with explanations for each hypothesized model.

**Hypothesis**	**Model diagram**	**Biological hypothesis**	**Hypothesis**	**Model diagram**	**Biological hypothesis**
1	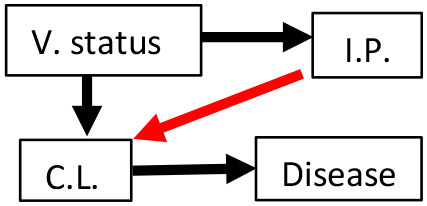	Immune parameter expression affects chlamydial load. Immune parameter expression and disease are unrelated.	4	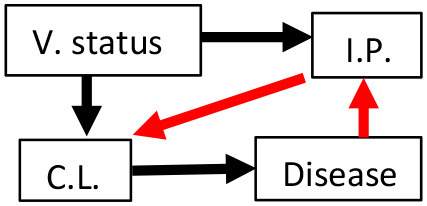	Immune parameter expression affects chlamydial load. Immune parameter expression up-regulated with disease.
2	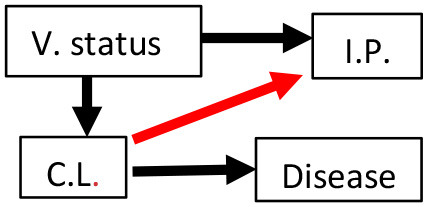	Chlamydial load affects immune parameter expression. Immune parameter expression and disease are unrelated.	5	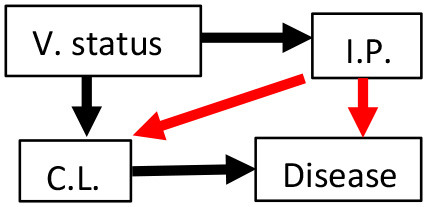	Immune parameter expression affects chlamydial load. Immune parameter expression leads to scarring and disease.
3	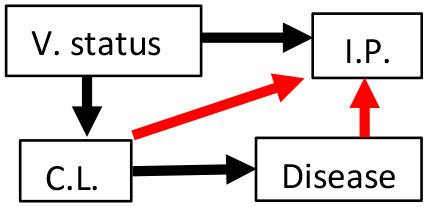	Chlamydial load affects immune parameter expression. Immune parameter expression up-regulated with disease.	6	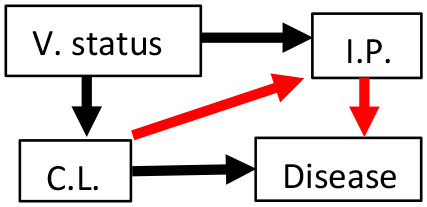	Chlamydial load affects immune parameter expression. Immune parameter expression leads to scarring and disease.

*Different models contain causal relationships assessed in different directions as indicated by the red arrows. The expression of one immune parameter was tested: interferon gamma (IFNg), or interleukin 17 (IL17). All six hypotheses were tested using chlamydial load and disease data collected from either the ocular or urogenital sites (i.e., 24 different models were tested in total). Arrows indicate the directionality of the relationship. Black arrows are relationships tested in only one direction, and red arrows indicate relationships tested in both directions*.

To begin, IFNγ or IL17 expression was evaluated as the immune parameter. Two models were made to determine if, (1) a causal relationship exists between these immune parameters and disease, and (2) the directionality between these immune parameters and chlamydial load (hypotheses 1 and 2). Four additional models were made to assess the directionality between (1) chlamydial load and these immune parameters, and (2) these immune parameters and disease (hypotheses 3, 4, 5, and 6). Only one immune parameter was included at a time in each of the six hypothesized models so as to maintain appropriate 10:1 sample size to variable ratios (36), resulting in 30 different models. The data were separated by either the urogenital or ocular site and tested in each hypothesized model, which resulted in a total of 60 models tested. The ocular models include ocular chlamydial load and disease status (e.g., ocular conjunctivitis), and the urogenital models include urogenital chlamydial load and disease status (e.g., cystitis or dilation of the ovarian bursae).

Prior to model testing, we chose to standardize data from all variables into a 0 to 1 scale to make all model coefficients between all variables comparable within each model. Binomial values were assigned to vaccination status (0 for non-vaccinated and 1 for vaccinated), and ocular and urogenital disease status (0 for clinical disease absent or subclinical and 1 for clinical disease present). Ocular and urogenital chlamydial load measurements were placed in one of three ordinal categories from 0 to 1 depending on an untransformed qPCR result similar to those used by Quigley et al. (30): (1) samples with no detectable qPCR result were given a value of 0, (2) samples with ≤ 100 copies·(μL of swab diluent tested)^−1^ were detectable but not quantifiable and given a value of 0.5, and (3) samples with > 100 copies·(μL of swab diluent tested)^−1^ were detectable and quantifiable and given a value of 1. Finally, values for both IFNγ and IL17 expression were transformed to a 0 to 1 scale by dividing each IFNγ or IL17 log fold expression measurement by the maximum IFNγ or IL17 log fold expression measurement, respectively, for all koalas in both studies.

Structural equation modeling (SEM) requires an individual to have all measurements for each variable tested in the model. We first tested each of the 60 models for model fit using the fit indices Root Mean Square Error of Approximation (RMSEA, absolute fit index) and Confirmatory Fit Index (CFI, relative fit index) described by Kline (37), and a Bollen-Stine Bootstrap for non-parametric model fit [100 bootstrap draws; (38, 39)]. No further analysis was performed for models that failed to fit the data using these indices [CFI > 0.9; RMSEA < 0.05; Bollen-Stine *p* > 0.05; (37, 38)]. Models that fit the data, however, were then compared using Akaike's information criterion corrected for small sample sizes (AICc) and model weights (w_i_) were calculated (40, 41). All models were tested using R statistical software [v3.5.3; (33)] using the lavaan package (42).

### Model Interpretation

A coefficient of determination (*R*^2^) was obtained for disease, chlamydial load, and immune parameter expression for each of the fitting models to estimate how well the model predicts each of these variables within the model. All standardized relationship coefficients (± 95% CI) were obtained for each path tested in each of the fitting models. Standardized relationship coefficients indicate the strength of the relationship (the magnitude of the coefficient), direction of the relationship (e.g., positive relationships indicate positive effects of one variable on another), and how well each relationship is predicted (by the 95% CI) by the model. We used standardized relationships in our model interpretation so we could compare the magnitude of all relationships within each model. As some models contained different sample sizes, care was taken not to compare relationship coefficients (or *R*^2^) across unrelatable models (i.e., models that do not contain the same variables). We defined strong model relationships based on 95% CI and whether it crossed 0, the threshold of an effect of an exogenous (predictor) variable on an endogenous variable (response). As our models were similar in complexity and limited by sample size, it was not uncommon that more than one model fit the data. For multiple model interpretation, we used model averaging to calculate model averaged parameters (and variance, 95% CI) using the previously described model weights [see Burnham et al. (41) for equation].

## Results

We first investigated the effects of MOMP vaccination 6 months after the initial vaccination on chlamydial abundance and disease at two important mucosal sites (ocular and urogenital) between two *Chlamydia* vaccination trials using free-ranging koalas (19, 20). We estimated effect sizes (as Hedge's g) for these responses [see Methods; (22)] and found no evidence for a difference in MOMP vaccination on chlamydial load (ocular and urogenital) and disease (urogenital) between field trials (meta-regression, *p* = 0.880; Figure 1A). A similar result was obtained when we analyzed the effect of MOMP vaccination on disease (urogenital) and transformed chlamydial load data (urogenital and ocular being placed into one of three ordinal categories, see Methods; meta-regression, *p* = 0.247). Estimations of heterogeneity were low for pairwise measurements of urogenital chlamydial load (*I*^2^ = 0.000), ocular chlamydial load (*I*^2^ = 0.000), and urogenital disease (*I*^2^ = 0.000). Additionally, we found measurements of IFNγ or IL17 expression in vaccinated animals to be comparable between the two experiments after performing a non-parametric Wilcoxon rank sum test (IFNγ, *p* = 0.936; IL17, *p* = 0.075; Figure 1B). As we found no evidence supporting a difference between the two trials, we pooled the data to obtain sample sizes sufficient to use in structural equation models.

The pooled data from the two trials resulted in a large collection of data for MOMP vaccinated (*n* = 50) or control (*n* = 46) wild koalas with recorded clinical disease status (control or vaccinated with ocular disease *n* = 1, control or vaccinated with urogenital disease *n* = 7; see Appendix 2) and measured chlamydial load out to 6 months post-vaccination. Of these 96 koalas, a subset of animals had measurements of either IFNγ (*n* = 40, *n* = 25 vaccinated koalas, *n* = 15 control koalas) or IL17 (*n* = 36, *n* = 25 vaccinated koalas, *n* = 11 control koalas) expression (see Appendix 2), and those that were missing these measurements were removed from the analysis (*n* = 51 and *n* = 55 for IFNγ and IL17, respectively). Of the 40 animals in the IFNγ expression subset (inclusive of all animals in the subset with IL17 expression), 13 animals had an ocular chlamydial load of ≥ 100 copies·μL^−1^ (*n* = 7 vaccinated, *n* = 6 control) at baseline, while 16 animals had an urogenital chlamydial load of ≥ 100 copies·μL^−1^ (*n* = 7 vaccinated, *n* = 9 control) at baseline. As sample size was limited for this analysis, we could not exclude animals with an infection at baseline nor could we introduce another variable into our models. We focussed on models with both immune parameters analyzed separately as structural equation modeling requires robust sample sizes [i.e., ratio of samples to variables analyzed = 10:1, (36); Appendix 3]. When we tested the 6 hypotheses for both the ocular and urogenital sites with either IFNγ or IL17 expression (Table 1), we obtained multiple models that fit the data (Appendix 4). For multiple hypothesis interpretation, we focussed on coefficients where the 95% confidence interval did not overlap 0 [a strategy used in other structural equation modeling studies such as Dorresteijn et al. (43)]. Additionally, we used a model averaging approach to determine if the direction and magnitude of these relationships were consistent across multiple models.

### Models With Ocular Chlamydial Load and Disease: MOMP Vaccination Reduced Ocular Chlamydial Load

**Figure 2:**
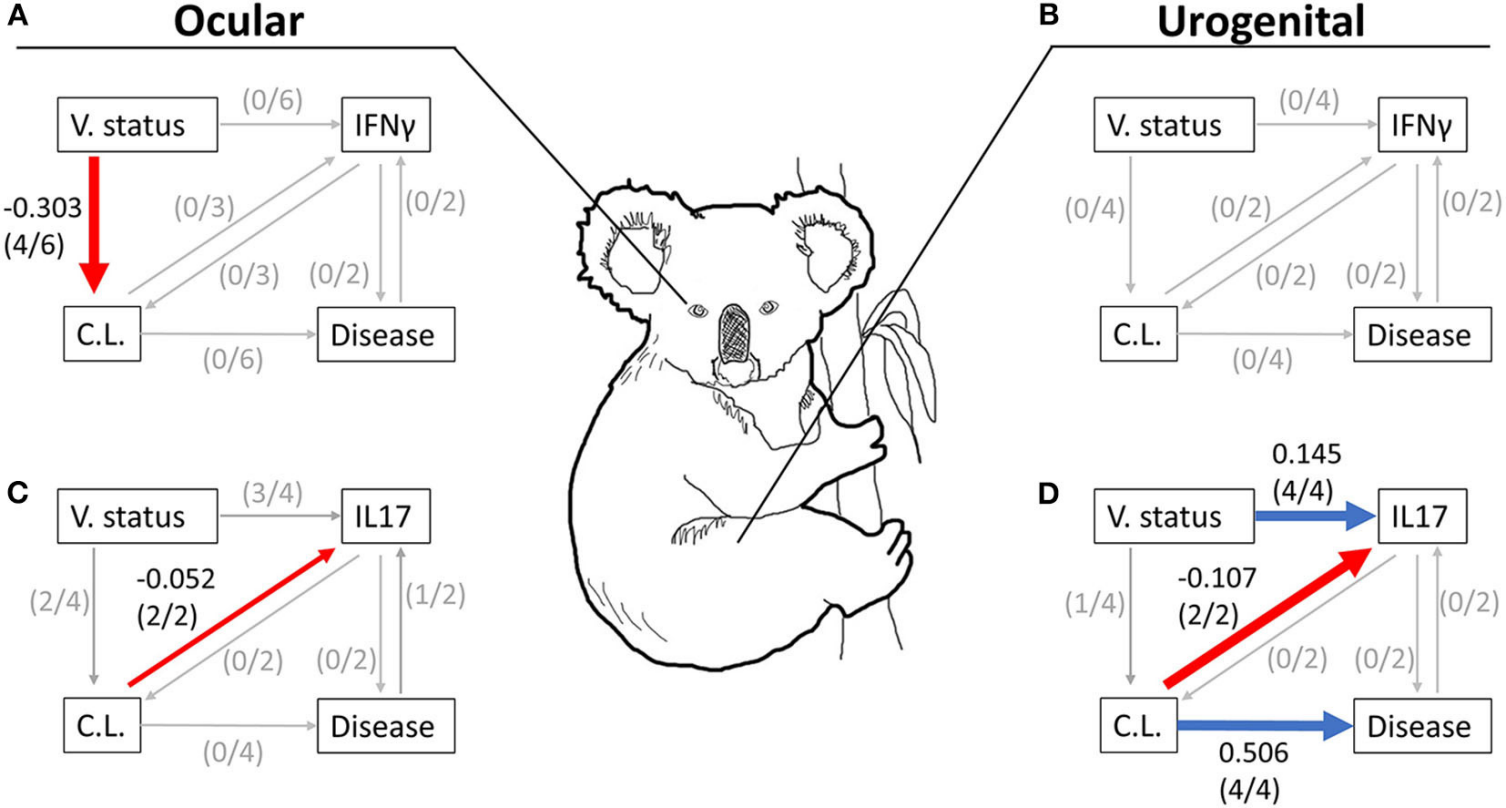
Summary figure of averaged models in Table 2 for ocular **(A,C)** and urogenital **(B,D)** data tested with either the expression of IFNγ **(A,B)** or IL17 **(C,D)** 6 months post-vaccination. Red and blue lines represent relationships with a negative or positive coefficient, respectively, where the coefficient and its variance (95% CI) did not cross 0 (i.e., the threshold of effect). Very small gray lines represent tested relationships in the averaged model where coefficients and its variance (95% CI) crossed 0. The number of hypotheses with a clear relationship (where the coefficient and its 95% CI did not cross 0) between two variables are listed in parentheses. Care should be made in the interpretation of this summary figure as it does not represent the results from any single model, but across all best fitting models using a model averaging approach. The directionality and magnitude of each coefficient shown in this figure are supported by significant coefficients (i.e., coefficients with 95% CIs that do not cross 0) obtained from the averaged models in Table 2. V. status, MOMP vaccination status; C.L., chlamydial load; Disease, chlamydial disease.

All six models with ocular chlamydial load, disease, and IFNγ expression fit the data (Appendix 4). We found that MOMP vaccination had a negative effect on chlamydial load in koalas (hypothesis 1, −0.286 ± 0.276; hyp. 2, −0.320 ± 0.300; hyp. 3, −0.320 ± 0.288; hyp. 6, −0.320 ±0.259; Table 2). We obtained the same result when all models were averaged together (model average, −0.303 ± 0.300; Table 2 and Figure 2A).

**Table 2:** Standardized estimates (± 95% CI) for each relationship within all best fitting models identified (see Appendix 4 for model fit and weights).

**Site**	**Immune parameter**	**Hypothesis/ Model Average**	**MVS → CL**	**MVS → IP**	**CL → Disease**	**IP → CL**	**CL → IP**	**Disease → IP**	**IP → Disease**
	IFNγ	1	**−0.286** **±** **0.276**	−0.065 ± 0.137	−0.043 ± 0.078	0.531 ± 30.064	–	–	–
		2	**−0.320** **±** **0.300**	−0.038 ± 0.084	−0.043 ± 0.080	–	0.083 ± 0.165	–	–
		3	**−0.320** **±** **0.288**	−0.039 ± 0.071	−0.043 ± 0.076	–	0.084 ± 0.135	0.016 ± 0.024	–
		4	−0.285 ± 0.547	−0.066 ± 11.25	−0.045 ± 0.304	0.538 ± 167.043	–	0.024 ± 574.97	–
		5	−0.286 ± 0.296	−0.065 ± 0.114	−0.044 ± 0.088	0.531 ± 41.195	–	–	0.007 ± 10.653
		6	**−0.320** **±** **0.259**	−0.038 ± 0.080	−0.044 ± 0.084	–	0.083 ± 0.155	–	0.007 ± 14.876
Ocular		Average	**−0.303** **±** **0.300**	−0.052 ± 0.659	−0.043 ± 0.091	0.266 ± 22.395	0.042 ± 0.081	0.002 ± 28.456	0.001 ± 1.250
	IL17	3	**−0.320** **±** **0.304**	**0.106** **±** **0.065**	−0.049 ± 0.080	–	−0.085 ± 0.089	**0.832** **±** **0.080**	–
		4	−0.253 ± 2.517	0.132 ± 8.159	0.010 ± 0.321	−0.402 ± 20.800	–	0.851 ± 296.338	–
		5	−0.257 ± 0.343	**0.166** **±** **0.104**	0.041 ± 0.057	−0.379 ± 0.388	–	–	0.526 ± 0.604
		6	–**0.320** **±** **0.314**	**0.130** **±** **0.100**	0.041 ± 0.057	–	–**0.115** **±** **0.102**	–	0.526 ± 0.570
		Average	−0.290 ± 0.846	0.131 ± 2.021	0.006 ± 0.129	−0.183 ± 5.092	–**0.052** **±** **0.052**	0.457 ± 71.095	0.240 ± 0.305
	IFNγ	3	−0.253 ± 0.296	−0.043 ± 0.080	0.347 ± 0.355	–	0.066 ± 0.108	0.116 ± 0.274	–
		4	−0.226 ± 0.251	−0.060 ± 0.106	0.303 ± 0.416	0.429 ± 38.306	–	0.120 ± 0.208	–
		5	−0.213 ± 0.251	−0.065 ± 0.135	0.267 ± 0.357	0.629 ± 26.074	–	–	0.645 ± 23.189
		6	−0.253 ± 0.261	−0.037 ± 0.096	0.267 ± 0.412	–	0.109 ± 0.216	–	0.645 ± 24.712
Urogenital		Average	−0.236 ± 0.265	−0.051 ± 0.104	0.298 ± 0.386	0.263 ± 16.549	0.043 ± 0.080	0.063 ± 0.130	0.299 ± 11.176
	IL17	3	**−0.320** **±** **0.261**	**0.112** **±** **0.084**	**0.394** **±** **0.361**	–	–**0.244** **±** **0.178**	0.208 ± 0.257	–
		4	−0.183 ± 0.300	**0.193** **±** **0.149**	**0.563** **±** **0.537**	−0.821 ± 0.841	–	0.241 ± 0.357	–
		5	−0.241 ± 0.343	**0.166** **±** **0.110**	**0.541** **±** **0.386**	−0.474 ± 0.541	–	–	0.688 ± 0.737
		6	−0.320 ± 0.321	**0.115** **±** **0.086**	**0.541** **±** **0.335**	–	–**0.161** **±** **0.122**	–	0.688 ± 0.792
		Average	−0.269 ± 0.308	**0.145** **±** **0.107**	**0.506** **±** **0.407**	−0.308 ± 0.398	–**0.107** **±** **0.084**	0.114 ± 0.161	0.338 ± 0.435

*MOMP vaccination status (MVS), immune parameter (IP; either IL17 or IFNγ expression), disease (either urogenital or ocular), and chlamydial load (CL; either urogenital or ocular) were modeled to test the six hypotheses described in Table 1. Dashed lines represent untested relationships. Bold values represent strong relationships where a coefficient ± 95% confidence interval does not cross zero*.

*Model averages are determined using the model weights in Appendix 4 (see Methods for model weight equation)*.

Four models containing ocular chlamydial load, disease, and IL17 expression fit the data (hypotheses 3, 4, 5, and 6; Appendices 4–6). Two of these hypotheses showed that koalas receiving a MOMP vaccination tended to have a lower chlamydial load (hypothesis 3, −0.320 ± 0.304; hyp. 6, −0.320 ± 0.314; Table 2 and Figure 2C), agreeing with models containing IFNγ expression. Three hypotheses indicate that MOMP vaccinated koalas produced more IL17 compared to control koalas (hypothesis 3, 0.106 ± 0.065; hyp. 5, 0.166 ± 0.104; hyp. 6, 0.130 + 0.100), and ocular chlamydial load negatively predicted IL17 expression (−0.115 ± 0.102; hypothesis 6). Lastly, one hypothesis supported a relationship between ocular disease and an increase in IL17 expression (0.832 ± 0.080, hypothesis 3; see Appendix 5 for ocular disease raw data); this however, is complicated as only one koala developed ocular disease after 6 months (IL17 fold expression compared to glyceraldehyde 3-phosphate dehydrogenase, GAPDH = 43.21, *n* = 1; see Appendix 5 for a comparison with other IL17 expression measurements). When all models were averaged, only one relationship had a marginal effect (ocular chlamydial load has an inverse relationship with IL17 expression; −0.052 ± 0.052; Table 2 and Figure 2C).

### Models With Urogenital Chlamydial Load and Disease: MOMP Vaccinated Koalas Reduced Chlamydial Load and Had More IL17 Gene Expression Compared to Control Animals

Four models with urogenital chlamydial load, disease, and IFNγ expression that fit the data (hypotheses 3, 4, 5, and 6; Appendices 4–6). When we investigated the coefficients for each relationship across all urogenital models with IFNγ expression, we found no clear relationships due to the large variance for each coefficient. As a result, we found no clear support for directionality or magnitude between IFNγ expression and urogenital chlamydial load or IFNγ expression and urogenital disease, though some relationships within these models were trending. We found a trending relationship between MOMP vaccinated koalas and a lower urogenital chlamydial load compared to control koalas (−0.236 ± 0.265, Table 2 and Figure 2B).

There were four hypotheses with urogenital chlamydial load and disease and IL17 expression that fit the data (hypotheses 3, 4, 5, and 6; Appendix 4). There was clear evidence that MOMP vaccinated koalas had a lower urogenital chlamydial load compared to control koalas (hypothesis 3, −0.320 ± 0.261). MOMP vaccinated koalas produced more IL17 compared to control animals that was supported by all four hypotheses (hypothesis 3, 0.112 ± 0.084; hyp. 4, 0.193 ± 0.149; hyp. 5, 0.166 ± 0.110; hyp. 6, 0.115 ± 0.086; Table 2). These four hypotheses also indicate that diseased koalas typically had a high chlamydial load (hypothesis 3, 0.394 ± 0.361; hyp. 4, 0.563 ± 0.537; hyp. 5, 0.541 ± 0.386; hyp. 6, 0.541 ± 0.335; Table 2). Two hypotheses indicate that when all koalas are considered (all 36 koalas from both the control or vaccinated groups) with a high chlamydial load tended to produce less IL17 compared to koalas with a low chlamydial load (hypothesis 3, −0.244 ± 0.178; hyp. 6, −0.161 ± 0.122; Table 2). Lastly, across all hypotheses, we found no relationship between IL17 expression and urogenital disease. When all urogenital models with IL17 expression were averaged we found: (1) that MOMP vaccinated koalas produced more IL17 compared to control koalas (0.145 ± 0.107), (2) koalas with a high chlamydial load were more likely to develop urogenital disease 6 months post-vaccination (0.506 ± 0.407), and (3) koalas (both vaccinated and control) with a high urogenital chlamydial load tended to express less IL17 mRNA compared to koalas with a lower urogenital chlamydial load (−0.107 ± 0.084; Table 2 and Figure 2D). These structural equation models therefore connect relationships among MOMP vaccination, IL17 expression, urogenital chlamydial load, and disease (Figure 2D).

## Discussion

There exist many unknown aspects surrounding chlamydial vaccination in koalas primarily due to the difficulty in obtaining samples and the cost for vaccine experiments. Much of the underlying chlamydial biology, particularly complex immunological responses to infections, is understood from experiments using the mouse model or from *in vitro* cell cultures. In the current study, data from two free-ranging koala vaccination trials (both using the major outer membrane protein, MOMP) were used to investigate the effects of MOMP vaccination both directly and indirectly on the expression of koala immune parameters, chlamydial infection, and disease. Koalas were compared from either vaccinated or control groups to evaluate vaccine efficacy given variability associated with free-ranging outbred animals (see Methods). Despite sources of variation in our data (numbers of koalas, use of outbred animals, unknown number of sexual encounters, unknown infection dates) there were clear relationships identified in our analyses that provide unique insights into the effects of vaccinating free-ranging koalas against *Chlamydia*, relative to unvaccinated individuals in the population. In particular, this study has helped establish one immune cytokine, IL17, may be an important factor in MOMP vaccinated koalas, however, animals with high chlamydial loads are expressing low amounts of IL17 and are more likely to develop urogenital disease.

Two measurements of the koala immune response were incorporated into the models created in this study, IFNγ and IL17 expression. IFNγ, a T-cell secreted cytokine, is currently regarded as one of most important cytokines in chlamydial biology (44). Across all pre-clinical studies investigating a chlamydial vaccine using various animal models (mice, non-human primates, cats, pigs, guinea pigs etc.), there exists a common aim of eliciting strong mucosal T-cell responses, particularly increases to IFNγ concentration (18, 22). Though IFNγ is involved in multiple immune pathways, IFNγ production leads to the enzymatic degradation of tryptophan, effectively starving *Chlamydia* of this essential amino acid (23). Some studies suggest that tryptophan starvation may even cause *C. trachomatis* to enter a persistence form (25, 45). Experiments conducted *in vitro* show that elevated IFNγ concentrations prevent chlamydial growth in *C. trachomatis*, but not *C. pecorum* (26). To our knowledge only one study, beside the two reported in the current study, has reported measurements of IFNγ expression and chlamydial load after MOMP vaccination in diseased koalas *ex vivo*. Nyari et al. (46) found that vaccination of captive koalas with MOMP (delivered along with Tri-adjuvant) did not have a clear effect on systemic IFNγ expression *ex vivo* despite a reduction in ocular disease among seven koalas (pre-vaccination compared to 6 weeks post-vaccination, *n* = 7). It is certainly possible that *C. trachomatis* and *C. pecorum* have differing sensitivities to IFNγ which may explain why measurements of koala IFNγ expression had no relationship with chlamydial load in any of our models (either ocular or urogenital; Table 2 and Figures 2A,B). These measurements of IFNγ expression contain large amounts of variance that can be seen in the raw data (Appendix 6). The average log transformed IFNγ measurement between the non-vaccinated and vaccinated groups were similar, 1.378 ± 0.918 (95% CI, *n* = 16) and 1.445 ± 0.430 (95% CI, *n* = 25), respectively. Additionally, IFNγ measurements have been shown to be dependent on the stage of chlamydial infection in mice (47) and non-human primates (48), something that is currently uncontrollable in trials using free-ranging koalas. More longitudinal studies are needed to determine if IFNγ expression is a key factor in protection against *C. pecorum* infection in free-ranging koalas.

In contrast, models with IL17 expression indicated that koalas that typically had higher ocular and urogenital chlamydial load tended to have low levels of IL17 expression, 6 months post-vaccination (Table 2 and red arrows in Figures 2C,D). Experiments using IL17 knockout mice have shown that IL17 is a factor in both protection (49) as well as pathogenesis (50). IL17 is a pro-inflammatory cytokine secreted mainly from T cells and has many functions in response to chlamydial infection. In the wild type mouse model and *in vitro* experiments, IL17 has been shown to work synergistically with IFNγ to inhibit *C. muridarum* growth by increasing the expression of inducible nitric oxide synthase (iNOS) and indirectly increasing antimicrobial nitric oxide [promoted by iNOS; (27)]. Indeed, iNOS has been shown to be up-regulated in human patients suffering from ectopic pregnancies associated with *C. trachomatis* infection (28). In koalas, one study reported the IL17a response from koala PBMCs to be higher in clinically diseased animals (*n* = 12) compared to subclinical animals [*n* = 29; (51)]. Three studies since have measured free-ranging koala IL17 response to chlamydial vaccination. In the first, Khan et al. (52) focussed on non-diseased koalas that tested negative for *Chlamydia*, finding that IL17 expression increased with MOMP vaccination when compared to pre-vaccination levels. The second and third, Waugh et al. (19) and Desclozeaux et al. (20), were included in our current analysis. One additional study using captive koalas with ocular disease reported comparable measurements of IL17a expression between koalas at pre-vaccination and 6 weeks post-vaccination (*n* = 7), even though there was improvement of ocular disease scores among the cohort at 6 weeks post-vaccination (46). In our current analysis, we found that koalas vaccinated with MOMP tended to have higher IL17 expression 6 months post-vaccination compared to non-vaccinated koalas in models based on urogenital data (Figure 2D). This was supported by three hypotheses (out of four) based on ocular data (Table 2). This increase can be seen by inspecting the raw data as the average log transformed expression of IL17 was higher in vaccinated compared to non-vaccinated groups, 1.616 ± 0.430 (95% CI, *n* = 25) and 0.462 ± 0.279 (95% CI, *n* = 12), respectively (Appendix 6). Additionally, we found no clear evidence for a direct relationship between IL17 expression and either ocular or urogenital disease that developed 6 months post-vaccination. Interestingly, the directionality of the relationship between chlamydial load and IL17, suggests that IL17 expression is dependent on chlamydial load. Perhaps animals with a high chlamydial load are unable to express IL17 or, more likely, are producing an immune response without IL17 when a high abundance of *Chlamydia* is present. Indeed, IL17 is one immune variable among many others that are part of a complex immune response to *Chlamydia* and its expression may or may not be a major factor in reducing chlamydial growth and preventing pathogenesis. More work needs to be done to determine if IL17 expression is linked to the reduction of chlamydial load and protection against disease in koalas.

Both Waugh et al. (19) and Desclozeaux et al. (20) evaluated the effect of MOMP vaccination on subsequent chlamydial disease, using univariate statistical methods. Waugh et al. (19) found that fewer koalas developed disease after 12 months in the MOMP vaccinated group (1 of 23 koalas) compared to control animals (4 of 27 koalas). This result, however, was not statistically significant (*X*^2^ = 1.512, *p* = 0.363) likely owing to the fact that many chlamydial infections remain subclinical and that the total sample size (*n* = 50) may have been too small to determine a statistically significant effect of vaccination on disease. Desclozeaux et al. (20) found that fewer koalas in the MOMP vaccinated group developed chlamydiosis (either ocular or urogenital chlamydial disease) compared to control koalas (0 of 21 and 3 of 21 for MOMP and control koalas, respectively); this comparison showed a trending (*p* < 0.1) effect between groups (*X*^2^ = 2.813, *p* = 0.093). Desclozeaux et al. (20) also found that six MOMP vaccinated koalas, with a detectable chlamydial infection recorded at pre-vaccination, reduced their chlamydial load (in either the ocular, urogenital or both sites) 6-months post-vaccination (6 of 21 koalas), and this effect was statistically significant compared to control koalas (0 of 21 koalas; *p* = 0.048).

By combining data from both trials, we were able to expand upon these analyses by introducing additional realistic complexity using structural equation models. Preliminary structural equation models indicated that a direct relationship between MOMP vaccination status and subsequent disease development failed our fitting criterion. Given this result, we created models where MOMP vaccination status indirectly affected disease status 6 months post-vaccination through direct relationships with either chlamydial load or an immune parameter. Ideally, we would have included both IFNγ and IL17 expression in the same models, but sample size limitations (having individual koalas in each group measured for all variables: IFNγ, IL17, infection load, disease status) prevented models with this level of complexity and, hence, we adopted a IFNγ or IL17 expression SEM approach. An additional ramification of our sample size limitation was that we were unable to exclude animals with baseline infections (animals with an ocular chlamydial load of >100 copies·μL^−1^, *n* = 7 vaccinated and *n* = 6 control; or urogenital chlamydial load of >100 copies·μL^−1^ *n* = 7 vaccinated and *n* = 9 control) nor were we able to include this as a variable within our models. Multiple models using ocular data and one model using urogenital data (Table 2) indicated that MOMP vaccination status had a direct negative effect on chlamydial load. These effects, however, were lost when we averaged all the urogenital models together. It is possible that given a larger sample size, we could have detected an effect. We performed a power analysis to determine the necessary sample size to detect a univariate effect of MOMP vaccination on urogenital chlamydial load. After estimating an effect size (Cohen's d) to estimate the effect of MOMP vaccination on urogenital chlamydial load (31), we found that a sample size of 117 koalas would be necessary to have an 80% chance of detecting an effect at a significance level of 0.05. This finding suggests that an effect might be obtained if a large-scale study included nearly three times the number of koalas that were used in our analysis.

In this study, we adopted a cross-sectional experimental design that focussed on control vs. vaccinated koalas at 6 months post-vaccination from which to implement our models. We chose this study design because research has shown koalas can become infected with *C. pecorum* and exhibit disease year-round. Therefore, background variation in individual parameters (immune factors, genotype, season sampled, etc.) appears insufficient to protect koalas from infection or disease, and the effect of a vaccine on immune parameters would most likely need to exceed normal background variation observed. We also acknowledge other valid study designs could be utilized. A common design for data similar to ours is to compare the change in variables over time (pre to post-vaccination) between the control and vaccinated groups. Although this is a valid approach, in our study system we chose not to adopt the change in variable approach for the reason described above, and because a vaccination effect (relative to controls) could be observed at the cohort level and still be within background variation. While not the determining reason, we do also acknowledge that utilizing a change in chlamydial load or host immune expression from baseline would further reduce the sample size for analyses (*n* = 3 for each model) in our study. Future research with a greater sample size of koala could consider a comparison of these designs, or the extent to which individual responses to vaccination over time are repeatable.

Chlamydial load is among one of several factors that affect disease pathogenesis in free-ranging koalas (30). Specifically, Quigley et al. (30) found that measurements of chlamydial load (separated into 3 ordinal categories of severity) are strong predictors of urogenital disease, but not ocular disease. In our current study, we also found that koalas that developed urogenital chlamydial disease had a higher chlamydial load as compared to non-diseased animals when the data were modeled with IL17 expression. We did not detect this effect in models of ocular chlamydial load and disease, though this was likely due to a low prevalence of koalas with ocular disease (*n* = 1). Previous studies have suggested that disease pathogenesis in koalas is complex (21, 53). Three out of four of our averaged models (Figures 2B–D) excluded Hypotheses 1 and 2, possibly suggesting that immune parameter and disease are related, however this remains unclear as only one relationship was significant (ocular chlamydial load and IL17; see Table 2). A large amount of variation in the prevalence of urogenital disease 6 months post-vaccination remains unexplained from even our best models (*R*^2^ = 0.268). Including more factors that are currently associated with disease pathogenesis, including koala retrovirus (KoRV) status, may improve the predictability of these models.

## Conclusions

In this study, we used structural equation models to identify direct and indirect relationships underpinning vaccination against chlamydial disease in free-ranging koalas. The use of structural equation modeling has previously been applied to identify complex factors influencing disease pathogenesis in koalas (30). One factor, urogenital chlamydial load, was positively associated with urogenital disease prevalence, agreeing with the findings of Quigley et al. (30). We found that MOMP vaccination may indirectly affect the development of urogenital disease in koalas by inhibiting the growth of *Chlamydia* (i.e., lower chlamydial load compared to control koalas). IL17 expression is one immune parameter that was increased in MOMP vaccinated koalas as compared to control koalas. Chlamydial load was a negative predictor of IL17 expression, suggesting that hosts express this cytokine less in response to chlamydial growth. Collectively, the structural equation models in this study highlight the importance of considering vaccine-immune-chlamydial load-disease relationships in a single analysis that can be performed in future studies of novel correlates of protection against *Chlamydia*. Lastly, more studies using free-ranging animals may be needed to determine the effect of multiple immune parameters on chlamydial load and disease and may make it possible to detect a direct effect of MOMP vaccination on chlamydial load.

## Data Availability Statement

Most of the data generated or analyzed during this study are included in the published articles (19, 20). Measurements of cytokine expression production for IL17 and IFNγ in the current study are available from the corresponding author on reasonable request.

## Ethics Statement

Collection of koala data previously collected by Waugh et al. (19) and Desclozeaux et al. (20) were approved by the University of the Sunshine Coast (USC) Animal Ethics Committee (AN/A/13/80) and the Queensland Government (Scientific Purposes Permit, WISP11532912).

## Author Contributions

DL, PT, BQ, and SC conceived the study design. PT and JH collected the data. DL performed the analysis. All authors contributed to interpreting the results and drafting of the manuscript.

## Conflict of Interest

JH was employed by the company Endeavor Veterinary Ecology. The remaining authors declare that the research was conducted in the absence of any commercial or financial relationships that could be construed as a potential conflict of interest.

## References

[1] Melzer A Carrick F Menkhorst P Lunney D John BS Overview, critical assessment, and conservation implications of koala distribution and abundance. Conserv Biol. (2000) 14:619–28. 10.1046/j.1523-1739.2000.99383.x

[2] McAlpine C Lunney D Melzer A Menkhorst P Phillips S Phalen D Conserving koalas: a review of the contrasting regional trends, outlooks and policy challenges. Biol Conserv. (2015) 192:226–36. 10.1016/j.biocon.2015.09.020

[3] Adams-Hosking C McBride MF Baxter G Burgman M De Villiers D Kavanagh R Use of expert knowledge to elicit population trends for the koala (*Phascolarctos cinereus*). Divers Distributions. (2016) 22:249–62. 10.1111/ddi.12400

[4] Rhodes JR Beyer H Preece H McAlpine C South east Queensland Koala Population Modelling Study. (2015). Available online at: https://environment.des.qld.gov.au/__data/assets/pdf_file/0029/88913/seq-koala-population-modelling-study.pdf (accessed September 12, 2020).

[5] Woinarski JC Burbidge AA Harrison PL. Ongoing unraveling of a continental fauna: decline and extinction of Australian mammals since European settlement. Proc Natl Acad Sci USA. (2015) 112:4531–40. 10.1073/pnas.1417301112 25675493 PMC4403217

[6] Mcalpine CA Bowen ME Callaghan JG Lunney D Rhodes JR Mitchell DL Testing alternative models for the conservation of koalas in fragmented rural–urban landscapes. Austral Ecol. (2006) 31:529–44. 10.1111/j.1442-9993.2006.01603.x

[7] Seabrook L McAlpine C Baxter G Rhodes J Bradley A Lunney D Drought-driven change in wildlife distribution and numbers: a case study of koalas in south west Queensland. Wildlife Res. (2011) 38:509–24. 10.1071/WR11064

[8] Lunney D Gresser S O'neill LE Matthews A Rhodes J The impact of fire and dogs on koalas at port stephens, new south wales, using population viability analysis. Pac Conserv Biol. (2007) 13:189–201. 10.1071/PC070189

[9] Rhodes JR Ng CF de Villiers DL Preece HJ McAlpine CA Possingham HP Using integrated population modelling to quantify the implications of multiple threatening processes for a rapidly declining population. Biol Conserv. (2011) 144:1081–8. 10.1016/j.biocon.2010.12.027

[10] McCallum H Kerlin DH Ellis W Carrick F Assessing the significance of endemic disease in conservation—koalas, *Chlamydia*, and koala retrovirus as a case study. Conserv Lett. (2018) 11:e12425 10.1111/conl.12425

[11] Beyer HL de Villiers D Loader J Robbins A Stigner M Forbes N Management of multiple threats achieves meaningful koala conservation outcomes. J Appl Ecol. (2018) 55:1966–75. 10.1111/1365-2664.13127

[12] Polkinghorne A Hanger J Timms P. Recent advances in understanding the biology, epidemiology and control of chlamydial infections in koalas. Vet Microbiol. (2013) 165:214–23. 10.1016/j.vetmic.2013.02.026 23523170

[13] Jackson M White N Giffard P Timms P. Epizootiology of *Chlamydia* infections in two free-range koala populations. Vet Microbiol. (1999) 65:255–64. 10.1016/S0378-1135(98)00302-2 10223324

[14] Jackson M Giffard P Timms P Outer membrane protein A gene sequencing demonstrates the polyphyletic nature of koala *Chlamydia pecorum* isolates. Syst Appl Microbiol. (1997) 20:187–200. 10.1016/S0723-2020(97)80065-3

[15] Loader J. An investigation of the health of wild koala populations in South-East Queensland. In: Bachelor of Applied Science (Animal Studies) Honours Thesis. St. Lucia, QLD: School of Animal Studies, The University of Queensland (2010). p. 1–219.

[16] Robbins A Loader J Timms P Hanger J. Optimising the short and long-term clinical outcomes for koalas (*Phascolarctos cinereus*) during treatment for chlamydial infection and disease. PLoS ONE. (2018) 13:e0209679. 10.1371/journal.pone.0209679 30589897 PMC6307739

[17] Craig AP Hanger J Loader J Ellis WA Callaghan J Dexter C. A 5-year *Chlamydia* vaccination programme could reverse disease-related koala population decline: Predictions from a mathematical model using field data. Vaccine. (2014) 32:4163–70. 10.1016/j.vaccine.2014.05.049 24877768

[18] Phillips S Quigley BL Timms P. Seventy years of *Chlamydia* vaccine research–limitations of the past and directions for the future. Front Microbiol. (2019) 10:70. 10.3389/fmicb.2019.00070 30766521 PMC6365973

[19] Waugh C Khan SA Carver S Hanger J Loader J Polkinghorne A. A prototype recombinant-protein based *Chlamydia pecorum* vaccine results in reduced chlamydial burden and less clinical disease in free-ranging koalas (*Phascolarctos cinereus*). PLoS ONE. (2016) 11:e0146934. 10.1371/journal.pone.0146934 26756624 PMC4710501

[20] Desclozeaux M Robbins A Jelocnik M Khan SA Hanger J Gerdts V. Immunization of a wild koala population with a recombinant *Chlamydia pecorum* major outer membrane protein (MOMP) or polymorphic membrane protein (PMP) based vaccine: new insights into immune response, protection and clearance. PLoS ONE. (2017) 126:e0178786. 10.1371/journal.pone.0178786 28575080 PMC5456371

[21] Madden D Whaite A Jones E Belov K Timms P Polkinghorne A. Koala immunology and infectious diseases: how much can the koala bear? Dev Comp Immunol. (2018) 82:177–85. 10.1016/j.dci.2018.01.017 29382557

[22] Lizárraga D Carver S Timms P. Navigating to the most promising directions amid complex fields of vaccine development: a chlamydial case study. Expert Rev Vaccines. (2019) 18:1323–37. 10.1080/14760584.2019.1698954 31773996

[23] Vasilevsky S Greub G Nardelli-Haefliger D Baud D. Genital *Chlamydia trachomatis*: understanding the roles of innate and adaptive immunity in vaccine research. Clin Microbiol Rev. (2014) 27:346–70. 10.1128/CMR.00105-13 24696438 PMC3993100

[24] Igietseme J Ukwade C Ouburg S Omosun Y Joseph K Profile of anti-*Chlamydia* immune responses in complicated (infertile) and non-complicated (fertile) genital infections. J Clin Immunol Immunother. (2015) 2:1–5. 10.24966/CIIT-8844/100006

[25] Beatty WL Belanger TA Desai AA Morrison RP Byrne GI. Tryptophan depletion as a mechanism of gamma interferon-mediated chlamydial persistence. Infect Immun. (1994) 62:3705–11. 10.1128/IAI.62.9.3705-3711.1994 8063385 PMC303021

[26] Islam MM Jelocnik M Huston WM Timms P Polkinghorne A. Characterization of the *in vitro Chlamydia pecorum* response to gamma interferon. Infect Immun. (2018) 86:e00714–17. 10.1128/IAI.00714-17 29358337 PMC5865032

[27] Zhang Y Wang H Ren J Tang X Jing Y Xing D. IL-17A synergizes with IFN-γ to upregulate iNOS and NO production and inhibit chlamydial growth. PLoS ONE. (2012) 7:e39214. 10.1371/journal.pone.0039214 22745717 PMC3379979

[28] Refaat B Al-Azemi M Geary I Eley A Ledger W Role of activins and inducible nitric oxide in the pathogenesis of ectopic pregnancy in patients with or without *Chlamydia trachomatis* infection. Clin Vaccine Immunol. (2009) 16:1493–503. 10.1128/CVI.00221-09 19692623 PMC2756840

[29] Agrawal T Bhengraj AR Vats V Salhan S Mittal A. Expression of TLR 2, TLR 4 and iNOS in cervical monocytes of *Chlamydia trachomatis*-infected women and their role in host immune response. Am J Reprod Immunol. (2011) 66:534–43. 10.1111/j.1600-0897.2011.01064.x 21883620

[30] Quigley BL Carver S Hanger J Vidgen ME Timms P. The relative contribution of causal factors in the transition from infection to clinical chlamydial disease. Sci Rep. (2018) 8:8893. 10.1038/s41598-018-27253-z 29891934 PMC5995861

[31] Borenstein M Hedges LV Higgens JP Rothstein HR. Introduction to Meta-Analysis. Hoboken, NJ: John Wiley & Sons, Ltd. (2009). p. 1–421. 10.1002/9780470743386

[32] Viechtbauer W Conducting meta-analyses in R with the metafor package. J Stat Softw. (2010) 36:1–48. 10.18637/jss.v036.i03

[33] Team RC: R: A Language and Environment for Statistical Computing Vienna: R Foundation for Statistical Computing (2017).

[34] Kollipara A George C Hanger J Loader J Polkinghorne A Beagley K. Vaccination of healthy and diseased koalas (*Phascolarctos cinereus*) with a *Chlamydia pecorum* multi-subunit vaccine: evaluation of immunity and pathology. Vaccine. (2012) 30:1875–85. 10.1016/j.vaccine.2011.12.125 22230583

[35] Hernández-Sánchez J Brumm J Timms P Beagley KW Vaccination of koalas with a prototype chlamydial vaccine is safe, does not increase the incidence of lymphoma-related disease and maybe associated with increased lifespan in captive koalas. Vaccine. (2015) 33:4459–63. 10.1016/j.vaccine.2015.07.029 26207589

[36] MacCallum RC Widaman KF Zhang S Hong S. Sample size in factor analysis. Psychol Methods. (1999) 4:84–99. 10.1037/1082-989X.4.1.8426822184

[37] Kline RB Principles and Practice of Structural Equation Modeling. New York, NY: Guilford publications (2015)

[38] Bollen KA Stine RA Bootstrapping goodness-of-fit measures in structural equation models. Sociol Methods Res. (1992) 21:205–29. 10.1177/0049124192021002004

[39] Kim H Millsap R. Using the bollen-stine bootstrapping method for evaluating approximate fit indices. Multiv Behav Res. (2014) 49:581–96. 10.1080/00273171.2014.947352 25558095 PMC4280787

[40] Burnham K Anderson D. Model Selection and Multi-Model Inference. Second NY: Springer-Verlag (2004). p. 63. 10.1007/b97636

[41] Burnham KP Anderson DR Huyvaert KP AIC model selection and multimodel inference in behavioral ecology: some background, observations, and comparisons. Behav Ecol Sociobiol. (2011) 65:23–35. 10.1007/s00265-010-1029-6

[42] Rosseel Y Lavaan: an R package for structural equation modeling and more. Version 0.5–12 (BETA). J Stat Softw. (2012) 48:1–36. 10.18637/jss.v048.i02

[43] Dorresteijn I Schultner J Nimmo DG Fischer J Hanspach J Kuemmerle T. Incorporating anthropogenic effects into trophic ecology: predator–prey interactions in a human-dominated landscape. Proc R Soc B Biol Sci. (2015) 282:20151602. 10.1098/rspb.2015.1602 26336169 PMC4571711

[44] Brunham RC Rey-Ladino J. Immunology of *Chlamydia* infection: implications for a *Chlamydia trachomatis* vaccine. Nat Rev Immunol. (2005) 5:149–61. 10.1038/nri1551 15688042

[45] Beatty WL Byrne GI Morrison RP. Morphologic and antigenic characterization of interferon gamma-mediated persistent *Chlamydia trachomatis* infection *in vitro*. Proc Natl Acad Sci USA. (1993) 90:3998–4002. 10.1073/pnas.90.9.3998 8387206 PMC46433

[46] Nyari S Booth R Quigley BL Waugh CA Timms P. Therapeutic effect of a *Chlamydia pecorum* recombinant major outer membrane protein vaccine on ocular disease in koalas (*Phascolarctos cinereus*). PLoS ONE. (2019) 14:e0210245. 10.1371/journal.pone.0210245 30615687 PMC6322743

[47] Cheng C Pal S Tifrea D Jia Z Luis M. A vaccine formulated with a combination of TLR-2 and TLR-9 adjuvants and the recombinant major outer membrane protein elicits a robust immune response and significant protection against a *Chlamydia muridarum* challenge. Microbes Infect. (2014) 16:244–52. 10.1016/j.micinf.2013.11.009 24291713 PMC3965591

[48] Cheng C Pal S Bettahi I Oxford KL Barry PA Luis M. Immunogenicity of a vaccine formulated with the *Chlamydia trachomatis* serovar F, native major outer membrane protein in a nonhuman primate model. Vaccine. (2011) 29:3456–64. 10.1016/j.vaccine.2011.02.057 21376796 PMC3084512

[49] Scurlock AM Frazer LC Andrews CW O'Connell CM Foote IP Bailey SL Interleukin-17 contributes to generation of Th1 immunity and neutrophil recruitment during *Chlamydia muridarum* genital tract infection but is not required for macrophage influx or normal resolution of infection. Infect Immun. (2011) 79:1349–62. 10.1128/IAI.00984-10 21149587 PMC3067500

[50] Andrew DW Cochrane M Schripsema JH Ramsey KH Dando SJ O'Meara CP. The duration of *Chlamydia muridarum* genital tract infection and associated chronic pathological changes are reduced in IL-17 knockout mice but protection is not increased further by immunization. PLoS ONE. (2013) 8:e76664. 10.1371/journal.pone.0076664 24073293 PMC3779189

[51] Mathew M Pavasovic A Prentis PJ Beagley KW Timms P Polkinghorne A. Molecular characterisation and expression analysis of Interferon gamma in response to natural *Chlamydia* infection in the koala, *Phascolarctos cinereus*. Gene. (2013) 527:570–7. 10.1016/j.gene.2013.06.019 23792018

[52] Khan SA Desclozeaux M Waugh C Hanger J Loader J Gerdts V. Antibody and cytokine responses of koalas (*Phascolarctos cinereus*) vaccinated with recombinant chlamydial major outer membrane protein (MOMP) with two different adjuvants. PLoS ONE. (2016) 11:e0156094. 10.1371/journal.pone.0156094 27219467 PMC4878773

[53] Wan C Loader J Hanger J Beagley K Timms P Polkinghorne A. Using quantitative polymerase chain reaction to correlate *Chlamydia pecorum* infectious load with ocular, urinary and reproductive tract disease in the koala (*Phascolarctos cinereus*). Austr Vet J. (2011) 89:409–12. 10.1111/j.1751-0813.2011.00827.x 21933169

[54] Lizárraga D Timms P Quigley BL Hanger J Carver S Capturing complex vaccine-immune-disease relationships for free-ranging koalas: IL17 production is an important intermediate factor against chlamydial disease. Res Square. (2020). 10.21203/rs.2.21243/v1 PMC7546032 33102563

[55] Lizárraga D Timms P Quigley BL Hanger J Carver S Capturing complex vaccine-775 immune-disease relationships for free-ranging koalas: IL17 production is an important 776 intermediate factor against chlamydial disease. ResearchSquare. (2020) 777 10.21203/rs.2.21243/v2 PMC7546032 33102563

