# Supplementary material for: Capturing Complex Vaccine-Immune-Disease Relationships for Free-Ranging Koalas: Higher Chlamydial Loads Are Associated With Less IL17 Expression and More Chlamydial Disease
Source: Front Vet Sci. 2020 Sep 25;7:530686. doi: 10.3389/fvets.2020.530686 (PMC7546032; doi:10.3389/fvets.2020.530686)
Supplement: Supplementary file 1 [file Data_Sheet_1.docx]

Appendix 1.

*Methods from Waugh et al. (2016) and Desclozeaux et al. (2017)*

Waugh et al. [1] prepared a vaccine consisting of purified recombinant *Chlamydia pecorum* major outer membrane protein (MOMP) genotypes A, F, and G (50 μg of each antigen) combined with Immunostimulating Complex (ISC) adjuvant (50 μg). Sixty animals were randomly assigned to either a vaccinated (n=30) or control group (n=30) in the study in the Moreton Bay Region, Queensland, Australia from 2012 to 2015. Vaccinated koalas received three subcutaneous vaccinations over three months at one-month intervals. All animals were assessed by a veterinarian before vaccination (i.e. day 0) and approximately six months post-vaccination for signs of chlamydial disease. During these assessments ocular and urogenital swabs were collected to detect *Chlamydia* by qPCR, and blood samples were collected to detect serum immunoglobulin G (IgG) or to measure the expression of interferon gamma (IFNγ) and interleukin 17 (IL17) using peripheral blood mononuclear cells (PBMCs) relative to the housekeeping gene glyceraldehyde 3-phosphate dehydrogenase (GAPDH). A single housekeeping gene, GAPDH, was used as the reference for gene expression as previous studies have shown that the expression of this gene remains relatively stable after stimulation with either mitogens [2,3] or chlamydial antigen (UV-inactivated *C. pecorum* G) [4,5]. The measurements of IFNγ and IL17 expression of mRNA were not published and the methods not described by Waugh et al. [1]. The methodology for measuring IFNγ and IL17 gene expression are, however, outlined in detail by Mathew et al. [4] for IFNγ and by Mathew et al. [5] for IL17. Both IFNγ and IL17 were normalised to GAPDH by the 2^-ΔΔCT^ method described by Livak and Schmittgen [6]: 2^-ΔΔCT^, where ΔΔCT is (threshold cycle (CT) for Cytokine expression – CT for GAPDH expression after 12 hours) – (CT for Cytokine expression – CT for GAPDH expression at time zero). Measurements of serum IgG were collected for 28 koalas (11 vaccinated and 17 control koalas), and an *in vitro* neutralisation assay was conducted for 9 control koalas. Due to the small number of koalas with measurements of IgG or *in vitro* neutralisation, we excluded these from further analysis in structural equation models (see Methods). Of the sixty koalas in this trial, 22 had both IFNγ and IL17 expression measurements. Additionally, 4 individuals had a measurement of IFNγ expression, but no measurement of IL17 expression (see Appendix 1a for the number of vaccinated and control groups). Of these subsets (26 and 22 koalas for IFNγ and IL17 expression, respectively), three of the control koalas and two of the vaccinated koalas had a urogenital disease diagnosis at six months post vaccination. There were no cases of ocular disease in either the vaccinated or control groups at six months post vaccination (see Appendix 1a).

The vaccine used by Desclozeaux et al. [7] consisted of purified recombinant major outer membrane protein (MOMP) genotypes A, F, and G (50 μg of each antigen) or peripheral membrane protein (PMP) genotype G, combined with tri-adjuvant consisting of PCEP (250 μg of poly[di(sodium carboxylatoethylphenoxy)]-phsophazene), IDR (500 μg), and polyI:C (250 μg). Sixty-three koalas from the Moreton Bay Region (different individuals from those vaccinated by Waugh et al. [1]) from 2013 to 2016 were randomly assigned to one of three groups: MOMP vaccinated (n=21), PMP vaccinated (n=21), or control (non-vaccinated; n=21). All animals were assessed by a veterinarian before vaccination (i.e. day 0) and approximately six months after the first vaccination for signs of chlamydial disease. Swabs were collected at the ocular and urogenital sites from all animals to determine chlamydial load by qPCR, but blood samples were only taken from vaccinated individuals for measurements of IFNγ and IL17 as previously described. Control animals from this trial did not have measurements of systemic cytokine expression. Of the 42 koalas in this trial that could be included in our analysis (i.e. PMP vaccinated individuals excluded), 14 vaccinated koalas had both IL17 and IFNγ expression measurements. There were no measurements of either IL17 or IFNγ expression for control koalas (see Appendix 1a). Of the 14 vaccinated koalas six months post vaccination, two koalas had a urogenital disease diagnosis and one koala had an ocular disease diagnosis.

Two individuals were included as controls in both the study by Waugh et al. [1] and Desclozeaux et al. [7] (Nyx and Teena). Both koalas were included only once in our study. We used the data reported by Waugh et al. [1] for these animals in our models (excluding any data for these koalas by Desclozeaux et al. [7]) as cytokine expression measurements were taken for control koalas.

*Biological Hypotheses*

In addition to the number of assumptions we made, we were interested in testing a number of hypothesized relationships between 1) host immune parameter and chlamydial load (hypotheses 1, 4, and 5), 2) chlamydial load and host immune parameter (hypotheses 2, 3, and 6), 3) immune parameter and disease (hypotheses 5 and 6), and 4) disease and host immune parameter (hypotheses 3 and 4). We chose hypotheses linking host immune parameter and chlamydial load (hypotheses 1, 4, and 5) as there is evidence that IFNγ is linked to chlamydial load reduction *in vitro* [8] and we hypothesized the expression of a host immune parameter would supress chlamydial load. We tested this hypothesis in the opposite direction (hypotheses 2, 3, and 6) to determine if animals having a small chlamydial abundance was a predictor of producing more of a given host immune parameter. We tested immune parameter and disease (hypotheses 5 and 6) as IL17 expression was previously hypothesized to be a cause of disease in koalas [5]. Lastly, we tested this relationship in the reverse direction (hypotheses 3 and 4) to determine if diseased koalas are more likely to upregulate host immune parameters.

Appendix 2. Study summaries of Waugh et al. 2016, Desclozeaux et al. 2017 and a total combining koalas or measurements from both studies. Pooled group sizes for structural equation modelling containing either IL17 or IFNγ mRNA expression include control and vaccinated koalas with complete data only (i.e. individuals with missing measurements were excluded). The bold numbers represent sample sizes for structural equation models in this study. MOMP = major outer membrane protein, PMP = peripheral membrane protein

| Study summary or group size | Waugh et al. 2016 | Desclozeaux et al. 2017 | Total |
| --- | --- | --- | --- |
| Years sampled (all seasons) | 2012 to 2015 | 2014 to 2016 | -- |
| Geographic location of trial | Moreton Bay, Queensland | Moreton Bay, Queensland | -- |
| Vaccine(s) tested | MOMP | MOMP, PMP | -- |
| Total number of koalas in trial | 60 | 63 | 123 |
| Number of unique MOMP vaccinated koalas | 30 | 21 | 51 |
| Number of unique control koalas | 30 | 19* | 51 |
| Cytokine measurements for vaccinated koalas? | Yes | Yes | -- |
| Number of koalas with an IL17 expression measurement (vaccinated) | 11 | 14 | 25 |
| Number of koalas with an IFNγ expression measurement (vaccinated) | 11 | 14 | 25 |
| Cytokine measurements for control koalas? | Yes | No | -- |
| Number of koalas with an IL17 expression measurement (control) | 11 | 0 | 11 |
| Number of koalas with an IFNγ expression measurement (control) | 15 | 0 | 15 |
| Pooled number of vaccinated and control koalas for a model with IL17 expression | 22 | 14 | **36** |
| Pooled number of vaccinated and control koalas for a model with IFNγ expression | 26 | 14 | **40** |
| Number of pooled vaccinated and control koalas with urogenital disease at 6 months post vaccination** | 5 | 2 | 7 |
| Number of pooled control and vaccinated koalas with ocular disease at 6 months post vaccination** | 0 | 1 | 1 |

*Two koalas (Nyx and Teena) were included as control koalas in both the trial by Waugh et al. 2016 and Desclozeaux et al. 2017. We included these individuals only once (included in the Waugh et al. 2016 column in the table), thus 19 individuals are reported in the column for Desclozeaux et al. 2017.
**Both pooled groups of vaccinated and control koalas that contain either IFNγ (n=40) or IL17 expression (n=36) have the same number of diseased individuals

Appendix 3. Sample size of MOMP-vaccinated koalas (data pooled for Waugh et al. 2016 and Desclozeaux et al. 2017) for each modelled variable and the number of koalas that have measurements for all variables (required for structural equation modelling) as models are incrementally increased in complexity. Two variable models can be analysed using single predictor generalized linear models, but would be less informative compared to structural equation models that require robust sample sizes. Bold terms are sample sizes used in models in this study. C.L. = chlamydial load; IFNγ = interferon gamma mRNA expression; IL17 = interleukin 17 mRNA expression

| Variables in model | Sample size for urogenital or ocular sites (n) | Suitable for SEM? |
| --- | --- | --- |
| Vaccination status, Disease | 93 | No* |
| Vaccination status, C.L. | 89 | No* |
| Vaccination status, IFNγ | 41 | No* |
| Vaccination status, IL17 | 37 | No* |
| **Vaccination status, Disease, C.L., IFNγ** | **40** | **Yes** |
| **Vaccination status, Disease, C.L., IL17** | **36** | **Yes** |
| Vaccination status, Disease, C.L., IFNγ, IL17 | 36 | No** |

*Use of a generalized linear model (GLM) is more suitable.
 **Insufficient sample size.

Appendix 4. Results of model fitting testing the six hypotheses shown in Table 1. Improvement in the model fit is listed as the change in Akaike’s information criterion with a correction for small sample sizes (ΔAICc) and model weight (w_i_), along with a coefficient of determination (R^2^) for disease, chlamydial load (C.L.), and expression of an immune parameter used in the model. Dashes lines indicate models that did not fit the data and were not compared using AICc.

| Site | Immune parameter | Hypothesis | Did the model fit? | ΔAICc | w_i_ | Disease R^2^ | C.L. R^2^ | Immune parameter R^2^ |
| --- | --- | --- | --- | --- | --- | --- | --- | --- |
| Ocular | IFNγ | 1 | Yes | 0.000 | 0.402 | 0.013 | 0.177 | 0.040 |
|  |  | 2 | Yes | 0.000 | 0.402 | 0.013 | 0.139 | 0.083 |
|  |  | 3 | Yes | 4.200 | 0.049 | 0.013 | 0.139 | 0.083 |
|  |  | 4 | Yes | 4.188 | 0.049 | 0.013 | 0.177 | 0.041 |
|  |  | 5 | Yes | 4.210 | 0.049 | 0.013 | 0.177 | 0.040 |
|  |  | 6 | Yes | 4.210 | 0.049 | 0.013 | 0.139 | 0.083 |
|  | IL17 | 1 | No | -- | -- | -- | -- | -- |
|  |  | 2 | No | -- | -- | -- | -- | -- |
|  |  | 3 | Yes | 0.000 | 0.304 | 0.014 | 0.137 | 0.536 |
|  |  | 4 | Yes | 0.477 | 0.240 | <0.001 | 0.164 | 0.503 |
|  |  | 5 | Yes | 0.575 | 0.228 | 0.456 | 0.174 | 0.123 |
|  |  | 6 | Yes | 0.575 | 0.228 | 0.456 | 0.137 | 0.161 |
| Urogenital | IFNγ | 1 | No | -- | -- | -- | -- | -- |
|  |  | 2 | No | -- | -- | -- | -- | -- |
|  |  | 3 | Yes | 0.077 | 0.263 | 0.125 | 0.180 | 0.101 |
|  |  | 4 | Yes | 0.000 | 0.273 | 0.123 | 0.157 | 0.157 |
|  |  | 5 | Yes | 0.324 | 0.232 | 0.188 | 0.162 | 0.040 |
|  |  | 6 | Yes | 0.324 | 0.232 | 0.188 | 0.101 | 0.106 |
|  | IL17 | 1 | No | -- | -- | -- | -- | -- |
|  |  | 2 | No | -- | -- | -- | -- | -- |
|  |  | 3 | Yes | 0.000 | 0.304 | 0.143 | 0.150 | 0.310 |
|  |  | 4 | Yes | 0.478 | 0.240 | 0.114 | 0.170 | 0.114 |
|  |  | 5 | Yes | 0.576 | 0.228 | 0.268 | 0.215 | 0.123 |
|  |  | 6 | Yes | 0.576 | 0.228 | 0.268 | 0.190 | 0.150 |

Appendix 5. Sample size for koala ocular chlamydial load placed in ordinal categories used in structural equation models (a), ocular disease status (b), and log transformed chlamydial load measurements were plotted against disease status (c). Light bars and unfilled symbols represent non-vaccinated koalas, and dark bars and filled symbols represent MOMP-vaccinated koalas. Chlamydial load qPCR values were grouped ordinally (based on untransformed measurements) such that samples with no detectable qPCR result were “not detectable”, samples with ≤100 copies·μL^-1^ were “detectable but not quantifiable”, and samples with >100 copies·μL^-1^ were “detectable and quantifiable”. Red lines indicate the mean value for each group.


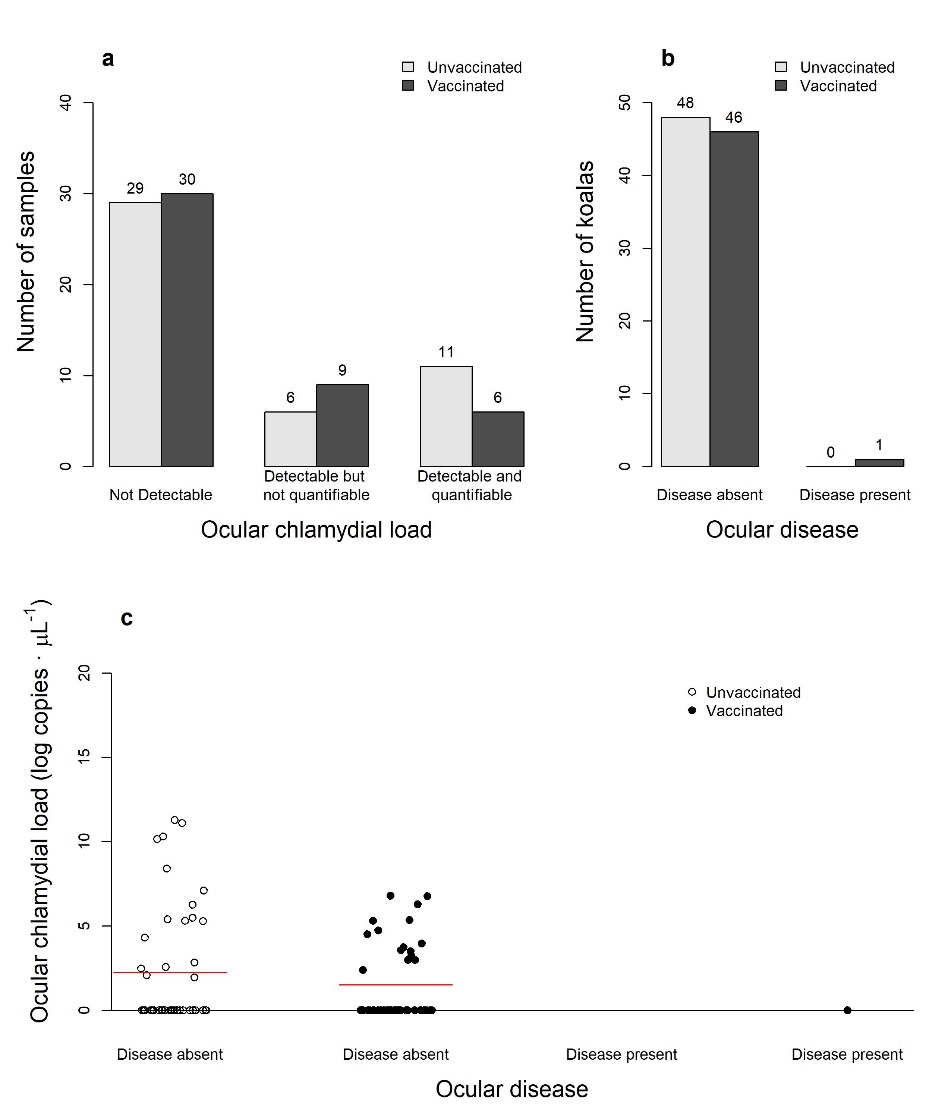


Appendix 6. Proportion of each log transformed fold gene expression value to the maximum log transformed fold gene expression value for both interferon γ (IFNg; circles) or interleukin 17 (IL17; triangles) in unvaccinated (unfilled symbols) or MOMP-vaccinated (filled symbols) free-ranging koalas (a) and IFNg expression (in both vaccinated and unvaccinated animals with a limited y-axis (one exceptional point is not visible) of individual IFNg expression values (b). Gene expression of IFNg or IL17 was measured from koala PBMCs collected six months post-vaccination. Cells were stimulated with *Chlamydia pecorum* elementary bodies and expression was compared to the housekeeping gene glyceraldehyde 3-phosphate dehydrogenase (GAPDH). Red lines indicate the mean value for each group.


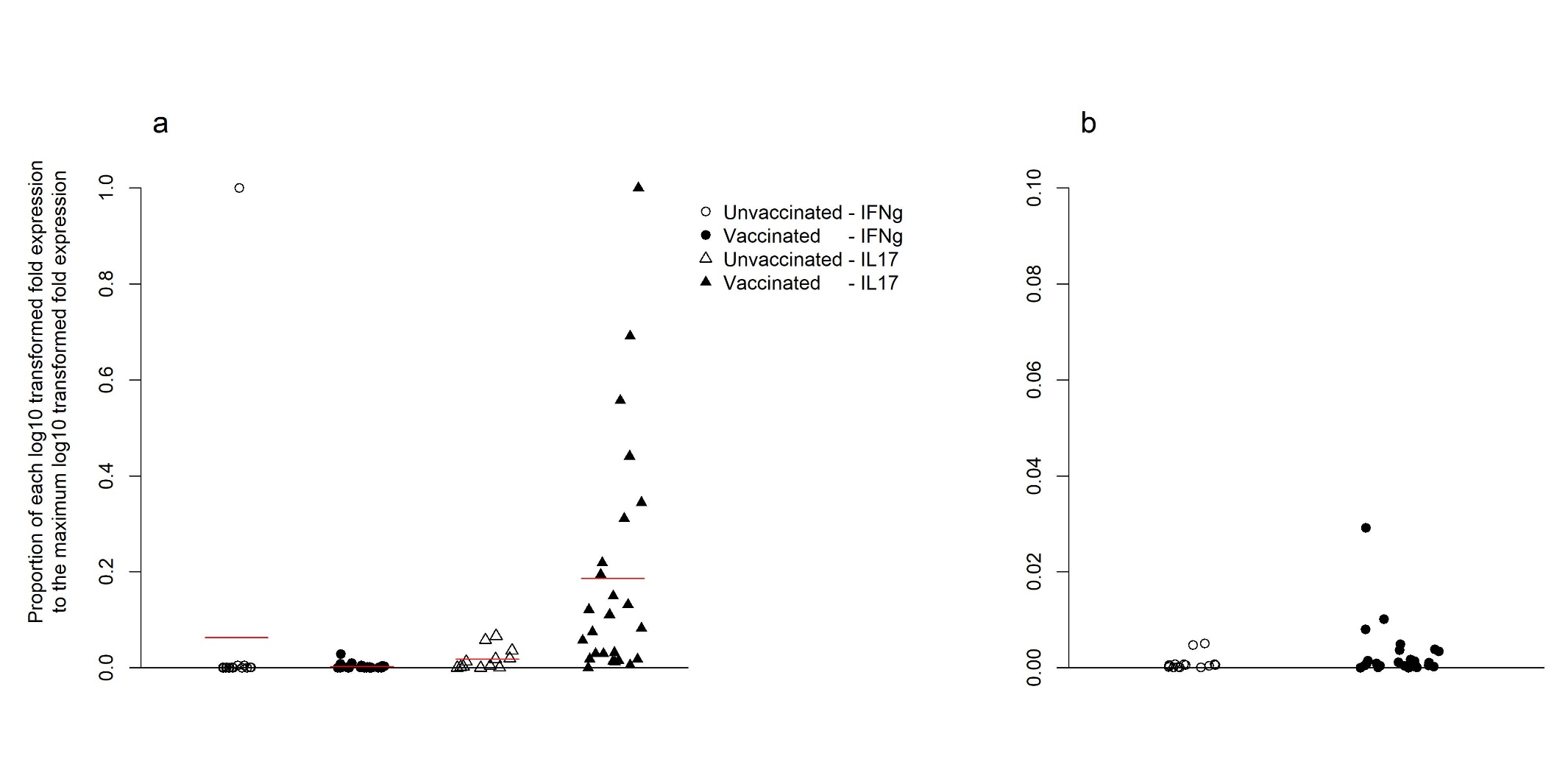


Appendix 7. Sample size for koala urogenital chlamydial load placed in ordinal categories used in structural equation models (a), urogenital disease status (b), and log transformed chlamydial load measurements were plotted against disease status (c). Light bars and unfilled symbols represent non-vaccinated koalas, and dark bars and filled symbols represent MOMP-vaccinated koalas. Chlamydial load qPCR values were grouped ordinally (based on untransformed measurements in copies·μL^-1^) such that samples with no detectable qPCR result were “not detectable”, samples with ≤100 copies·μL^-1^ were “detectable but not quantifiable”, and samples with >100 copies·μL^-1^ were “detectable and quantifiable”. Red lines indicate the mean value for each group.


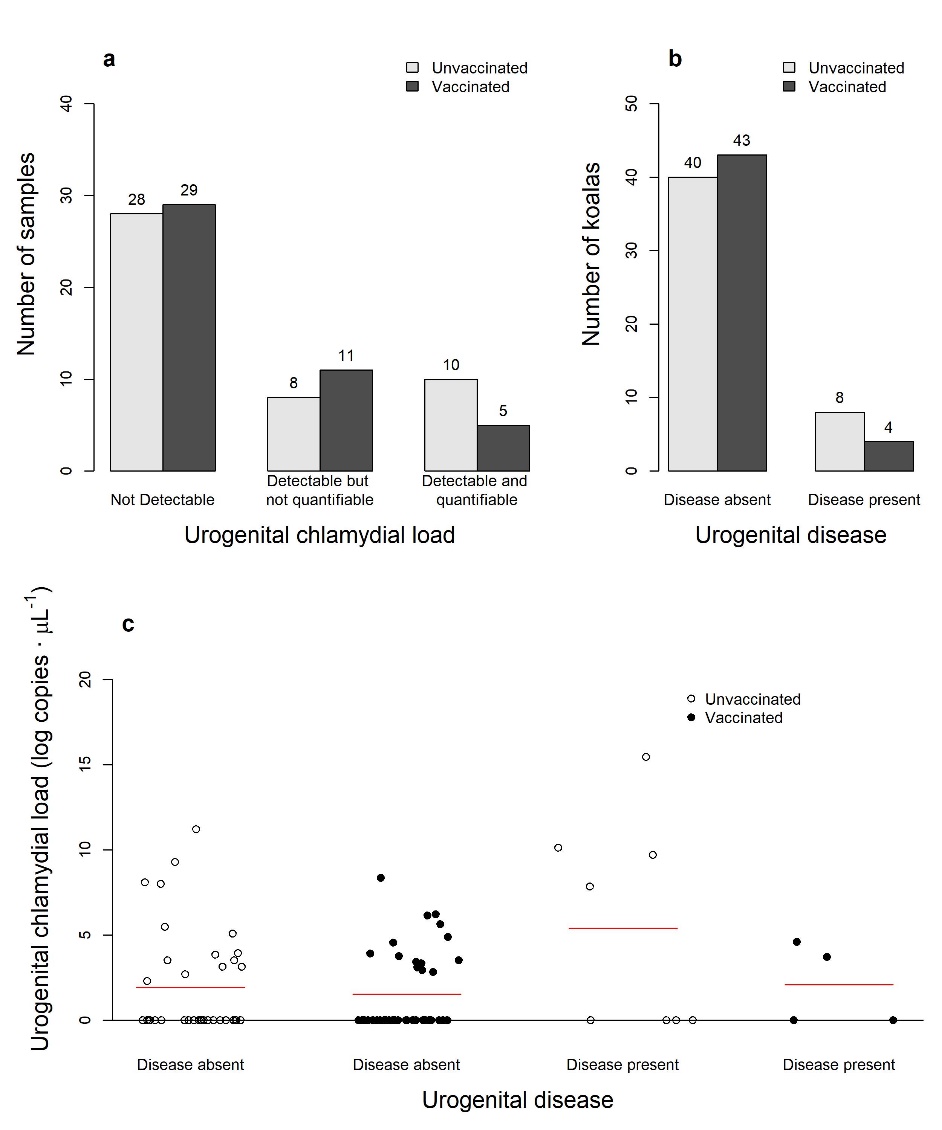


References

1. Waugh C, Khan SA, Carver S, Hanger J, Loader J, Polkinghorne A, et al. A prototype recombinant-protein based *Chlamydia pecorum* vaccine results in reduced chlamydial burden and less clinical disease in free-ranging koalas (*Phascolarctos cinereus*). PloS one. 2016;11 1:e0146934.

2. Maher IE, Griffith JE, Lau Q, Reeves T, Higgins DP. Expression profiles of the immune genes CD4, CD8β, IFNγ, IL-4, IL-6 and IL-10 in mitogen-stimulated koala lymphocytes (*Phascolarctos cinereus*) by qRT-PCR. PeerJ. 2014;2 280:1-19.

3. Sarker N, Fabijan J, Emes RD, Hemmatzadeh F, Meers J, Moreton J, Owen H, Seddon JM, Simmons G, Speight N and Trott D. Identification of stable reference genes for quantitative PCR in koalas. Scientific reports. 2018;8 1:1-8.

4. Mathew M, Pavasovic A, Prentis PJ, Beagley KW, Timms P, Polkinghorne A. Molecular characterisation and expression analysis of Interferon gamma in response to natural *Chlamydia* infection in the koala, *Phascolarctos cinereus*. Gene. 2013;527 2:570-7.

5. Mathew M, Waugh C, Beagley KW, Timms P, Polkinghorne A. Interleukin 17A is an immune marker for chlamydial disease severity and pathogenesis in the koala (*Phascolarctos cinereus*). Developmental and Comparative Immunology. 2014;46 2:423-9; doi: 10.1016/j.dci.2014.05.015. <Go to ISI>://WOS:000340216600033.

6. Livak KJ, Schmittgen TD. Analysis of relative gene expression data using real-time quantitative PCR and the 2− ΔΔCT method. Methods. 2001;25 4:402-8.

7. Desclozeaux M, Robbins A, Jelocnik M, Khan SA, Hanger J, Gerdts V, et al. Immunization of a wild koala population with a recombinant *Chlamydia pecorum* Major Outer Membrane Protein (MOMP) or Polymorphic Membrane Protein (PMP) based vaccine: New insights into immune response, protection and clearance. PloS One. 2017;12 6:e0178786.

8. Beatty WL, Byrne GI, Morrison RP. Morphologic and antigenic characterization of interferon gamma-mediated persistent *Chlamydia trachomatis* infection *in vitro*. Proceedings of the National Academy of Sciences. 1993;90 9:3998-4002.
